# Supplementary figures and images for: Shared input and recurrency in neural networks for metabolically efficient information transmission
Source: PLoS Comput Biol. 2024 Feb 23;20(2):e1011896. doi: 10.1371/journal.pcbi.1011896 (PMC10917264; doi:10.1371/journal.pcbi.1011896)

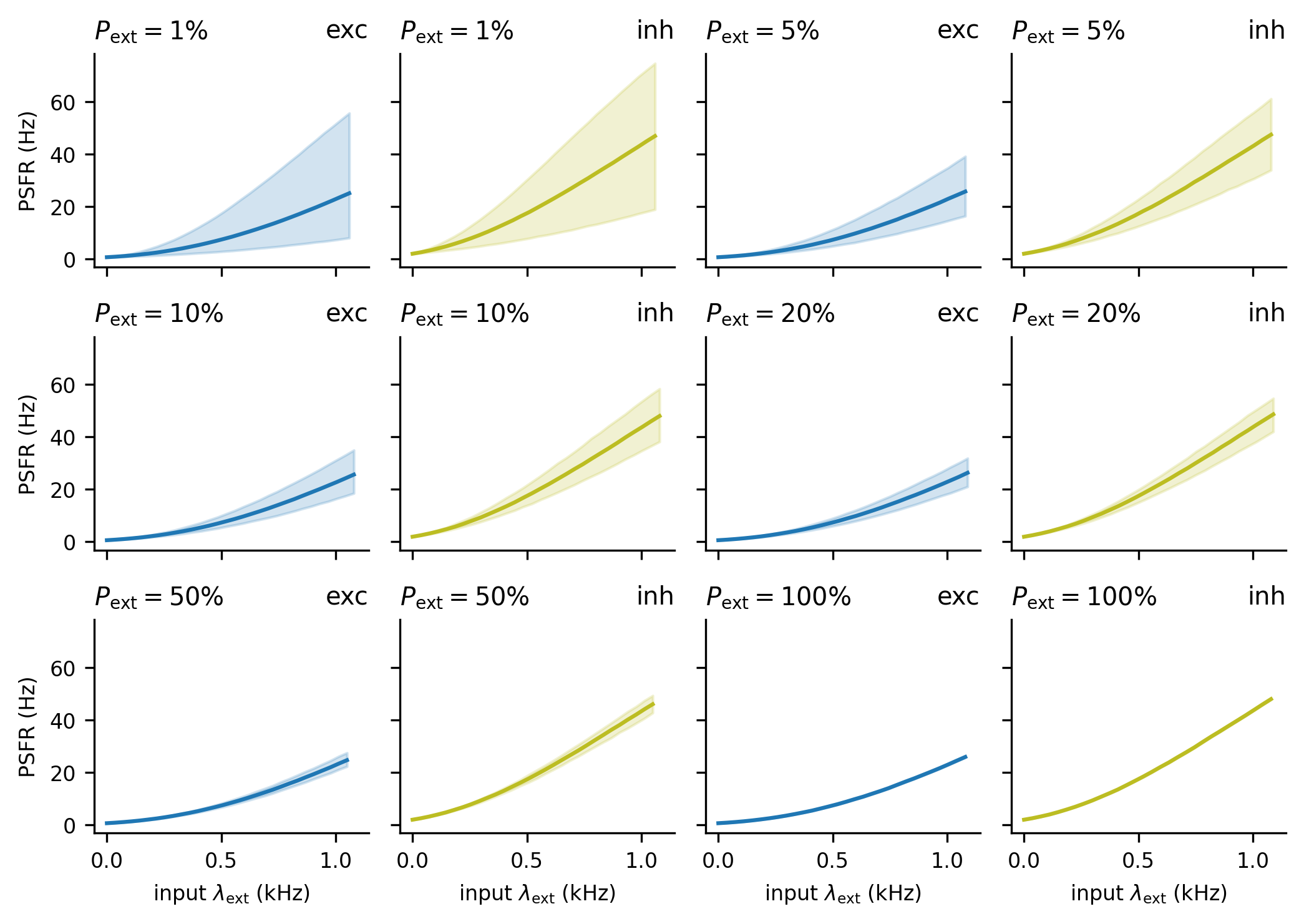

Supplement: S1 Fig — To exclude the network effects, we plotted the tuning curves for the feedforward network separately for the excitatory (blue) and inhibitory (yellow) neurons. The thick line represents the median response across the neurons, which shows that their tuning curves are convex in the studied range. The shaded area shows the spread of the tuning curves across neurons (2.5 to 97.5 percentile). With low values of Pext, the tuning curves across neurons vary significantly and are skewed to the higher firing rates. (TIF) [file pcbi.1011896.s001.tif]

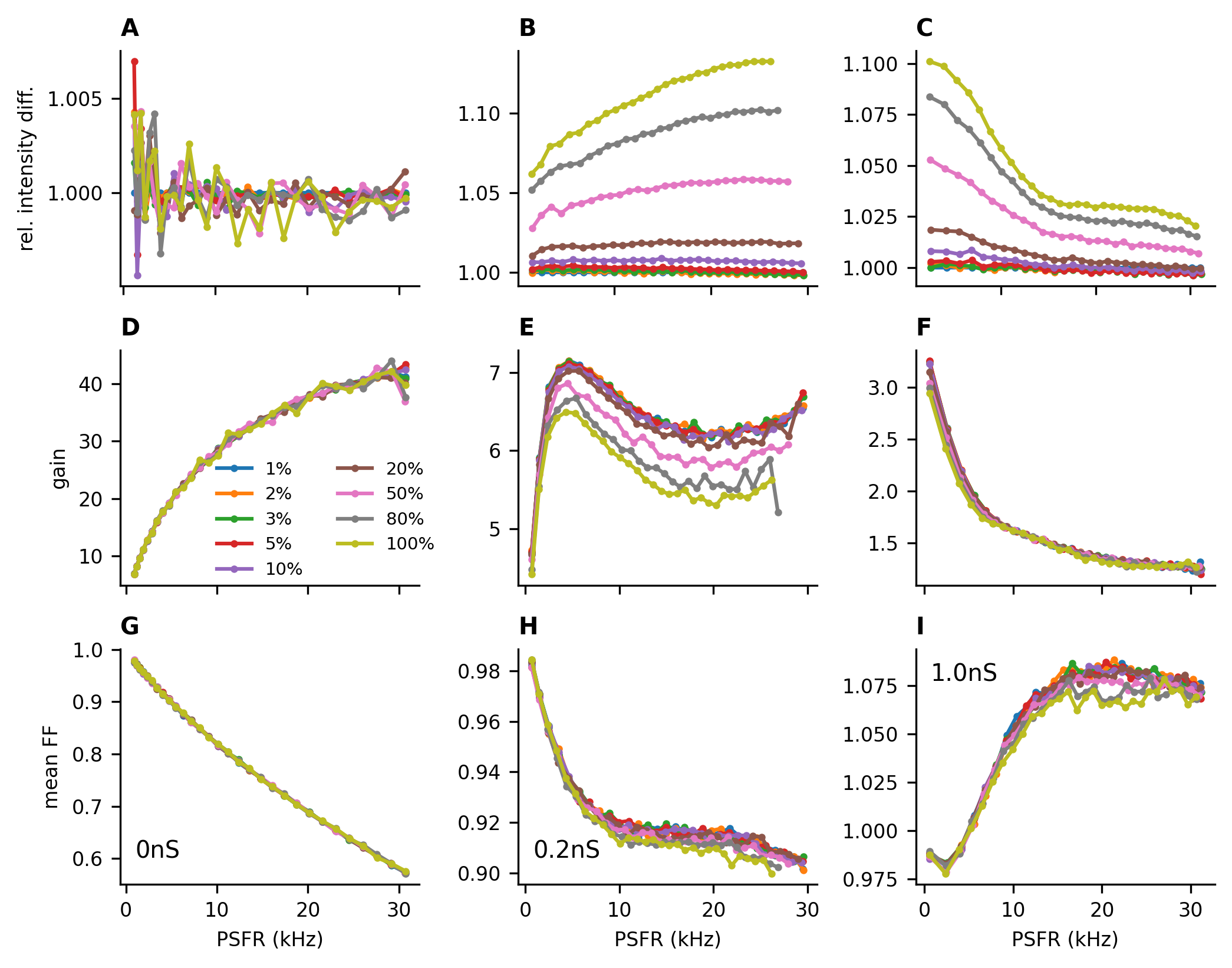

Supplement: S2 Fig — Same as Fig 4, but exactly kextPext external neurons connected to each excitatory and inhibitory neuron. This removed a large part of the dependence on Pext seen in Fig 4. (TIF) [file pcbi.1011896.s002.tif]

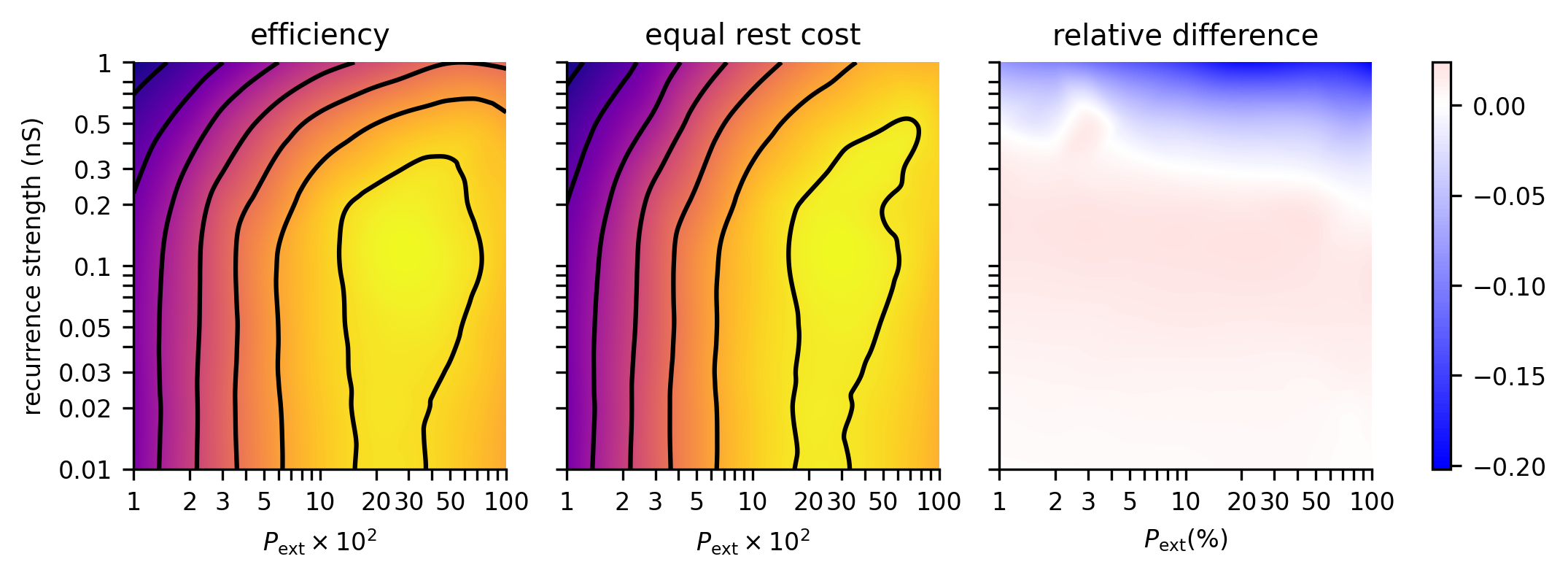

Supplement: S3 Fig — We observed that the cost of the resting state was different for different recurrence strengths arec (Fig 3A–3C). This could potentially explain the higher information-metabolic efficiency E (Eq 10) for intermediate values of arec and its decrease for high values of arec. To quantify the effect of the resting cost, we set the resting cost in each case to the resting cost of the feedforward network W0(arec = 0). The differences in the cost of the resting state did not have a qualitative effect on the conclusions. A: The same contour plot as in Fig 5B. B: Contour plot with equalized resting costs (contours as in Fig 5B: 0.75, 1.0, 1.25, 1.5, 1.75, 2.0, and 2.25 bits/s). C: Heatmap of the relative differences. (TIF) [file pcbi.1011896.s003.tif]

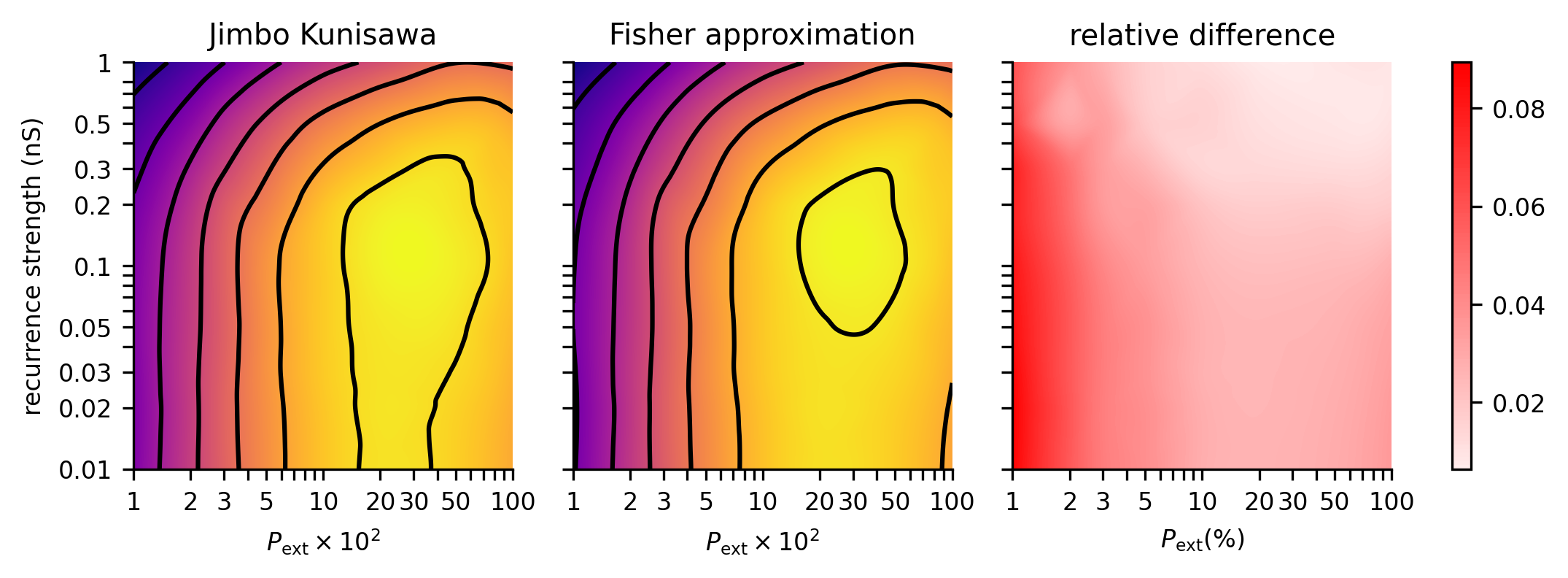

Supplement: S4 Fig — To calculate the capacity-cost functions, we calculated the mutual information using Eq (5) with the input probability distribution calculated from Eqs (12) and (14). Here we compare the information-metabolic efficiencies calculated with the approximation and the Jimbo-Kunisawa algorithm. A: The same contour plot as in Fig 5B with information-metabolic efficiencies calculated with the Jimbo-Kunisawa algorithm. B: Information-metabolic efficiencies calculated with the Fisher-information-based input distribution. C: Heatmap of the relative differences. Note that the approximation can only reach values lower than the actual information-metabolic efficiency. (TIF) [file pcbi.1011896.s004.tif]

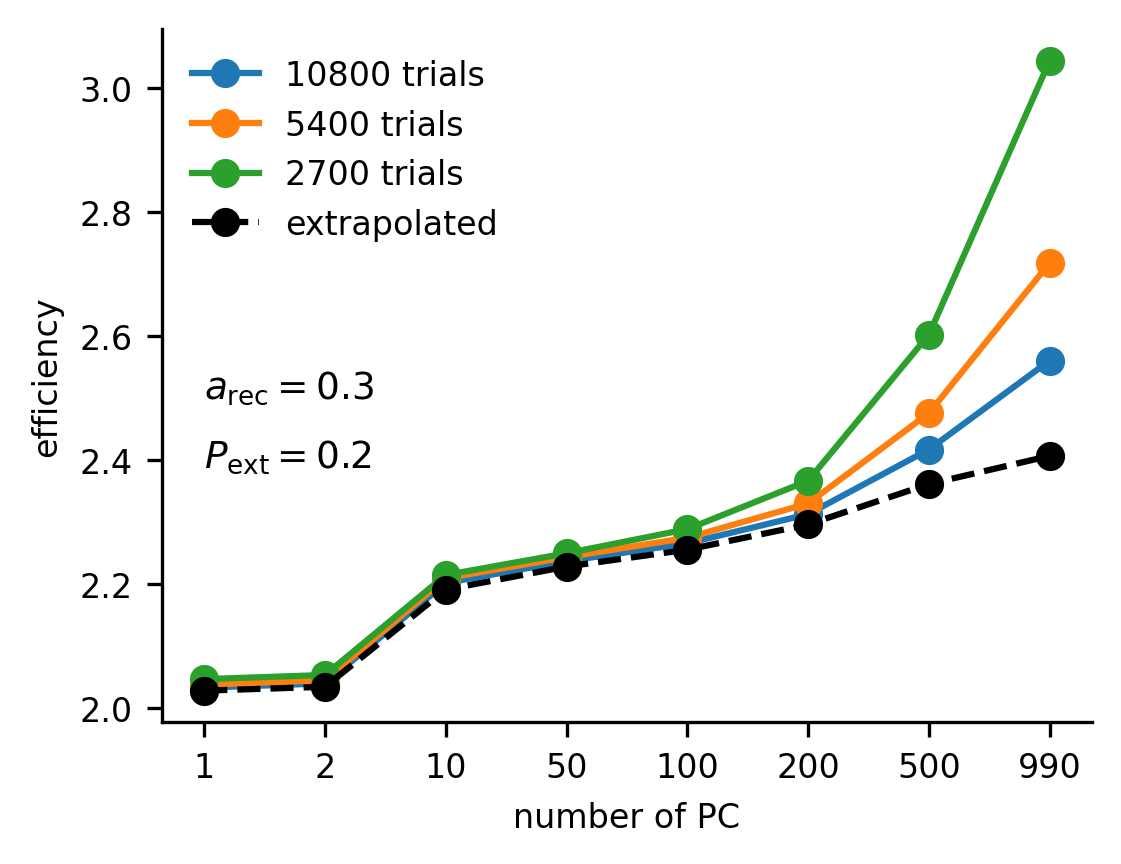

Supplement: S5 Fig — The information-metabolic efficiency calculated by the Jimbo-Kunisawa algorithm is plotted for different numbers of principal components used. We calculated the information-metabolic efficiency from different numbers of trials. At high number of components, lower number of trials lead to significantly higher information-metabolic efficiency. This is the effect of the sampling bias. We attempted to remove the bias by using the quadratic extrapolation method. For 500 principal components the bias is still relatively low, and increasing the number of components brings little benefit in terms of information-metabolic efficiency. (TIF) [file pcbi.1011896.s005.tif]
