# Supplementary material for: Shared input and recurrency in neural networks for metabolically efficient information transmission
Source: PLoS Comput Biol. 2024 Feb 23;20(2):e1011896. doi: 10.1371/journal.pcbi.1011896 (PMC10917264; doi:10.1371/journal.pcbi.1011896)
Supplement: Attachment — Submitted filename: Plos comp bio revision Barta Kostal May 2023 (2).pdf [file pcbi.1011896.s006.pdf]

Barta and Kostal analyze a network of leaky integrate and fire consisting of excitatory and inhibitory neurons that receive external inputs from a uniform population of Poisson neurons. Under the assumption that information is read out by summing (or averaging) the activity of all the neurons in the network, the authors analyze the trade-off between information transfer and metabolic costs. I like the ideas and the formalization of the paper and I believe it could be, upon improvement, published in this journal. Nonetheless, I have some high-level concerns/questions, some comments on the structure & accessibility of the paper, plus a list of minor issues that I believe could improve the clarity and accessibility of the paper.

High-level comments:

1) **Biological plausibility 1; external inputs.** The paper analyzes the situation where all the external neurons have exactly the same (Poisson) statistics and connect to a proportion of ex/inh neurons. I understand that the authors want to analyze a simple model, but this choice seems to be at odds with the brain. In the introduction, the authors say that they model “a small portion of the sensory cortex and the noise correlations that arise from an external thalamic population”. Can we think of the output of the thalamus as a homogenous population of Poisson neurons? If yes, could the authors provide references for that, and explicitly mention in the title and in the abstract that this model is valid only for this regime? In the present title & abstract, it sounds like the paper’s conclusions hold for general brains and networks. On the other side, I think it could be very biologically relevant to extend the model a bit and consider heterogeneous external populations where neurons have tuning functions for one or more variables. In that case, the conclusions of the paper could be much more general and important for the computational neuroscience field.

2) **Biological plausibility 2; network readout.** The authors assume that the readout of the network is a simple summation (or an average) of the population activity. This seems to be at odds with many neuroscientific findings, where the entire population vector (or a projection thereof onto a low-D space) carries information about the input. Although the summed population activity can carry information, that can be related to other aspects of neural dynamics (e.g., oscillations, sleep/wake cycles, neuromodulation, other physiological aspects, etc..). It is possible that, in the specific case analyzed by the authors (the small portion of sensory cortex), downstream areas indeed read information by averaging or summing. If that is the case, could the authors make this clear in the abstract and introduction, and provide references for that?

3) **Noise correlations.** The authors analyze noise correlations in the simple case where the external neurons are uniform, and the information is measured between external avg. firing rate and summed network output. In that situation, I believe that the conclusions in the papers cited by the authors (Abbot and Dayan 1999, Averback 2006, Moreno-Bote 2014), i.e. that noise correlations are detrimental for information transfer, apply for this setting. Nonetheless, apart from the mainstream arguments, there are other works where noise correlations can positively impact the transfer of information (see e.g. a recent review from Stefano Panzeri, Monica Moroni, Houman Safaai, Christopher D Harvey for an overview, but there are others). It would be nice if the authors took a more neutral stance towards noise correlations, also considering

that their results, in the end, suggest that some levels of noise correlations actually arise in the optimal solution. Moreover, if the authors decided to explore the situation where the input neurons have specific tuning, that would offer the possibility to explore relationships between input-tuning similarity vs noise correlations among pairs of cells. In that situation, theoretical work (Gasper Tkacik, Jason S Prentice, Vijay Balasubramanian, Elad Schneidman, 2010) suggests that the transfer of information and the beneficial vs. detrimental effects of noise correlations strongly depend on the SNR of the inputs, making it far from obvious that noise correlations necessarily hinder information transfer.

Paper structure and comprehensibility

4) **methods vs results.** It is not clear if the authors assume that readers will read the methods before reading the results. In the latter case, it is hard to understand some of the analyses that the authors performed. For example, section 3.1 is clear and understandable. The switch from a linear system to the spiking neural network is then not very clear in section 3.2, and it would be nice to have a very small overview of the network used without necessarily having to go to the methods. Certain sections then feel hard to understand and follow logically, with results described a bit rashly or with slightly obscure introductions and explanations. This might be OK for specialists, but it won't be enough for people who are not very familiar with these topics. As an example, take section 3.6: the authors write "Similarly, the increased probability of a synapse from an external neuron to a neuron in the excitatory or inhibitory population decreases the cost of neural activity but increases the trial-to-trial variability of the population response by increasing the noise correlations." This sentence: 1) is a bit too long 2) is hard to parse, as there are several logical implications 3) doesn't have references to previous results 4) sounds like a general statement about the brain or RNNs, although it actually refers to Fig. 2 and is valid only in this specific modeling case & choices (there are other scenarios where the opposite might be true). I kindly ask the authors to consider rephrasing some parts of the results to make them more accessible and understandable for a general audience.

5) **choice of parameters and assumptions.** The paper relies on many choices of parameters, both for the model and for the analyses, and it is not always clear how these parameters were fixed. Although the authors provide some references in the methods, many times it is unclear if the choice is related to previous work, if it was a pondered choice, or if it doesn't matter. For example, the authors fitted a 7th-degree polynomial function to the mean output of the network as a function of the stimulus (section 2.2): where does the 7th degree come from? Was there a statistical procedure involved? Or is this based on previous work? This is only one example, but there are some others throughout the paper that could leave the reader perplexed. I would be happy to see some effort toward explaining the modeling and parameter choices.

6) **Derivation of the metabolic cost of neural activity.** Section 2.3 provides some details on the calculations the authors followed to determine ATP expenditures. The derivation is fairly formal, but there are two passages that are a bit more hand-wavy: a) "The minimal number of Na ions is the quadrupled to get a more realistic estimate

[...]" and b) "cost is 1.25^10^8 ATP. However, about 80% of the cost associated with an action potential is expected to come from the propagation [...], therefore, we estimate the total cost as 6.25 X 10^8 ATP" (so essentially you multiplied by 5). Both these passages might be plausible, but in the current form of the manuscript they seem to be not well justified. Are they based on previous works or calculations? Also, how much are the final results influenced by these estimates? It seems to me that this is a crucial calculation but I'd be happy to see some analyses on that.

7) **Generally hard to understand for non specialists.** As suggested in several parts above, the text is sometimes not clear. Moreover, the authors try to use common words to explain mathematical concepts (e.g. trial-to-trial variability to refer to the fano factor), but this sometimes leads to confusion as there is no clear concept of "trials" in this study. More on this in points 8c and 9a,b below.

<u>Minor points</u>

8) Methods:
    a) Section 2.1, what does the subscript "bcg" stand for?
    b) Section 2.2, it would be nice to start with a small description of what the authors want to achieve before jumping into the calculations. This section of the methods I found not very clear.
    c) Section 2.4.1, the authors start by saying "If the trial-to-trial variability is very low,..." without introducing what they mean with that.
9) Text:
    a) Section 3.2, the authors mention that they measured pairwise correlations, but it is not clear on what time window that was done. The authors mention a dt=1sec in the methods, what the same dt used here? If a reader reads the results first, they might be left wondering about those important details.
    b) Section 3.3, as already said above, it is not 100% clear what the authors refer to with trial-to-trial variability. Is each dt considered a trial? If yes, it would be helpful to spell this out in the text.
    c) Section 4, at the beginning of the discussions, the authors claim that "populatons in the cortex that transmit information by their summed activity can be thought of as low noise information channels". Although I believe this is true in theory, it would be helpful to know if this strategy is employed in practice by the brain (refer to comment 2 above).
10) Figures
    a) Fig 1, panel B) it'd be useful to have a title for the colorful legend, and it would be useful to invert the scale (i.e. start from high at the top to low at the bottom). Also, are the two subplots related to excitatory and inhibitory cells?
    b) Fig 1, panel B) & F), Fig 2, Fig 3E), Fig 4, Fig 5A): it'd be useful to have a title for the colorful legends so as to avoid needing to search for that in the figure caption.
