# Supplementary material for: Shared input and recurrency in neural networks for metabolically efficient information transmission
Source: PLoS Comput Biol. 2024 Feb 23;20(2):e1011896. doi: 10.1371/journal.pcbi.1011896 (PMC10917264; doi:10.1371/journal.pcbi.1011896)
Supplement: Attachment — Submitted filename: optimal_coupling___response.pdf [file pcbi.1011896.s008.pdf]

We would like to thank the reviewers for their careful reading and positive evaluation of our work, and we apologize for the long time it took us to finish the revision. We addressed all the comments of the reviewers in the new version of the manuscript. We believe that incorporating these comments helped significantly to improve our manuscript. We think the manuscript is now easier to understand and more coherent. In particular, in order to address the second comment of Reviewer 1, we came up with an approach to efficiently estimate the mutual information between the one dimensional output and d-dimensional output. This way we did not have to limit ourselves to a single-dimensional readout of the summed activity. The high-dimensional output also required us to significantly increase the number of trials ($10\times$), leading to less noise in some of the figures. Below we provide a point-by-point response to individual comments of the reviewers:

Response to Referee 1

High-level comments

1. ***Biological plausibility 1; external inputs.*** *The paper analyzes the situation where all the external neurons have exactly the same (Poisson) statistics and connect to a proportion of ex/inh neurons. I understand that the authors want to analyze a simple model, but this choice seems to be at odds with the brain. In the introduction, the authors say that they model "a small portion of the sensory cortex and the noise correlations that arise from an external thalamic population". Can we think of the output of the thalamus as a homogeneous population of Poisson neurons? If yes, could the authors provide references for that, and explicitly mention in the title and in the abstract that this model is valid only for this regime? In the present title and abstract, it sounds like the paper's conclusions hold for general brains and networks. On the other side, I think it could be very biologically relevant to extend the model a bit and consider heterogeneous external populations where neurons have tuning functions for one or more variables. In that case, the conclusions of the paper could be much more general and important for the computational neuroscience field.*

   We agree that it would be very interesting to generalize our work by considering external population with heterogeneous tuning functions, such as tuning to orientation. However, such analysis would likely require a much larger network. Our studied population could for example only contain a fraction of neurons from a single barrel in the rat barrel cortex. Such a small number of neurons is likely to be tuned to the same stimulus. We now comment on this in the manuscript. We believe that such efforts could be feasible, using for example a similar approach as was used for modeling mesoscopic populations of neurons in the visual cortex, as done by (Zerlaut et al., 2017). Moreover, we are pleased that our approach to estimate the mutual information from a population of neurons (see below), instead of their summed activity, which we present in the new version of the manuscript, could be easily generalized for this purpose. However, the full implementation of the reviewer's suggestion is out of scope of the present work.

2. ***Biological plausibility 2; network readout.*** *The authors assume that the readout of the network is a simple summation (or an average) of the population activity. This seems to be at odds with many neuroscientific findings, where the entire population vector (or a projection thereof onto a low-D space) carries information about the input. Although the summed population activity can carry information, that can be related to other aspects of neural dynamics (e.g., oscillations, sleep/wake cycles, neuromodulation, other physiological aspects, etc..). It is possible that, in the specific case analyzed by the authors (the small portion of sensory cortex), downstream areas indeed read information by averaging or summing. If that is the case, could the authors make this clear in the abstract and introduction, and provide references for that?*

   Reading information by averaging or summing is essentially one of the simplest decoders the downstream areas could implement. In the new version of our manuscript, we decided to study the other extreme case, where we assume that the downstream areas act as an efficient unbiased decoder, i.e., achieving the Cramér–Rao bound. We calculate the mutual information between the input and the first 10 principal component. While we found minor improvements (10% to 20%) in the information-metabolic efficiency of the system, the key message is that the results remain qualitatively the same.

3. ***Noise correlations.*** *The authors analyze noise correlations in the simple case where the external neurons are uniform, and the information is measured between external avg. firing rate and summed network output. In that situation, I believe that the conclusions in the papers cited by the authors (Abbot and Dayan 1999, Averback 2006, Moreno-Bote 2014), i.e. that noise correlations are detrimental for information transfer, apply for this setting. Nonetheless, apart from the mainstream arguments, there are other works where noise correlations can positively impact the transfer of information (see e.g. a recent review from Stefano Panzeri, Monica Moroni, Houman Safaai, Christopher D Harvey (Panzeri et al., 2022) for an overview, but there are others). It would be nice if the authors took a more neutral stance towards noise correlations, also considering that their results, in the end, suggest that some levels of noise correlations actually arise in the optimal solution. Moreover, if the authors decided to explore the situation where the input neurons have specific tuning, that would offer the possibility to explore relationships between input-tuning similarity vs noise correlations among pairs of cells. In that situation, theoretical work (Tkačik et al., 2010) suggests that the transfer of information and the beneficial vs. detrimental effects of noise correlations strongly depend on the SNR of the inputs, making it far from obvious that noise correlations necessarily hinder information transfer.*

   As suggested, we are now more neutral about noise correlations. Particularly, we tried to clearly explain when the noise correlations are detrimental and why this applies to the case studied in our manuscript.

4. ***Methods vs Results.*** *It is not clear if the authors assume that readers will read the methods before reading the results. In the latter case, it is hard to understand some of the analyses that the authors performed. For*

example, section 3.1 is clear and understandable. The switch from a linear system to the spiking neural network is then not very clear in section 3.2, and it would be nice to have a very small overview of the network used without necessarily having to go to the methods. Certain sections then feel hard to understand and follow logically, with results described a bit rashly or with slightly obscure introductions and explanations. This might be OK for specialists, but it won't be enough for people who are not very familiar with these topics. As an example, take section 3.6: the authors write "Similarly, the increased probability of a synapse from an external neuron to a neuron in the excitatory or inhibitory population decreases the cost of neural activity but increases the trial-to-trial variability of the population response by increasing the noise correlations." This sentence: 1) is a bit too long 2) is hard to parse, as there are several logical implications 3) doesn't have references to previous results 4) sounds like a general statement about the brain or RNNs, although it actually refers to Fig. 2 and is valid only in this specific modeling case and choices (there are other scenarios where the opposite might be true). I kindly ask the authors to consider rephrasing some parts of the results to make them more accessible and understandable for a general audience.

We moved Methods to the end of the manuscript and rephrased the Results to be accessible without the need to keep referring to Methods. We also tried to frame our results better to avoid any confusion about in which conditions our conclusions apply.

5. ***Choice of parameters and assumptions.*** *The paper relies on many choices of parameters, both for the model and for the analyses, and it is not always clear how these parameters were fixed. Although the authors provide some references in the methods, many times it is unclear if the choice is related to previous work, if it was a pondered choice, or if it doesn't matter. For example, the authors fitted a 7th-degree polynomial function to the mean output of the network as a function of the stimulus (section 2.2): where does the 7th degree come from? Was there a statistical procedure involved? Or is this based on previous work? This is only one example, but there are some others throughout the paper that could leave the reader perplexed. I would be happy to see some effort toward explaining the modeling and parameter choices.*

   We added more justifications and references to parameter choices. Regarding the degree of the polynomial, t was purely our observation that 7-th degree polynomial captures well the course of the function without producing substantial oscillations. To avoid any confusion, we now use linear interpolation.

6. ***Derivation of the metabolic cost of neural activity.*** *Section 2.3 provides some details on the calculations the authors followed to determine ATP expenditures. The derivation is fairly formal, but there are two passages that are a bit more hand-wavy: a) "The minimal number of Na ions is the quadrupled to get a more realistic estimate [...]" and b) "cost is* 1.25×10^8 *ATP. However, about 80% of the cost associated with an action potential is expected to come from the propagation [...], therefore, we estimate the total cost as* 6.25×10^8 *ATP" (so essentially you multiplied by*

5). Both these passages might be plausible, but in the current form of the manuscript they seem to be not well justified. Are they based on previous works or calculations? Also, how much are the final results influenced by these estimates? It seems to me that this is a crucial calculation but I'd be happy to see some analyses on that.

We understand that from our text it might not have been clear that we did not come up with this calculation. We referenced in this part of the calculation again the work by Attwell (Attwell and Laughlin, 2001) (section Energy needed for action potentials). This was also confirmed by calculations taking the dendritic morphology into account (Vetter et al., 2001). We also now reference this work in our calculations. We note that we erroneously used the 4-fold value as the cost of propagation of the action potential and therefore multiplied the cost by 5, when instead the original estimate only multiplies the cost by 4. This does not have a qualitative effect on the results and in the new version of the manuscript we multiply the cost by 4 instead of 5. In a new supplementary figure, we illustrate that there are no qualitative differences if the cost of action potential is multiplied by 3, 4, or 5.

7. ***Generally hard to understand for non specialists.*** *As suggested in several parts above, the text is sometimes not clear. Moreover, the authors try to use common words to explain mathematical concepts (e.g. trial-to-trial variability to refer to the Fano factor), but this sometimes leads to confusion as there is no clear concept of "trials" in this study. More on this in points 8c and 9a,b below.*

   We hope to have made the text significantly easier to understand by incorporating the comments from both reviewers. Trial-to-trial variability, specifically, is now only used once, when we define the Fano factor and use Fano factor only further on.

Minor points

9. *Methods:*

   a) *Section 2.1, what does the subscript "bcg" stand for?*

      We now explain in the text in stands for the background activity.

   b) *Section 2.2, it would be nice to start with a small description of what the authors want to achieve before jumping into the calculations. This section of the methods I found not very clear.*

      In order to make the text easier to read, we moved the Methods at the end of the manuscript and we provide more explanations and definitions in the Results.

   c) *Section 2.4.1, the authors start by saying "If the trial-to-trial variability is very low,..." without introducing what they mean with that.*

      We replaced trial-to-trial variability throughout the text with Fano factor.

10. *Text:*

a) *Section 3.2, the authors mention that they measured pairwise correlations, but it is not clear on what time window that was done. The authors mention a dt=1sec in the methods, what the same dt used here? If a reader reads the results first, they might be left wondering about those important details.*

This is the correct interpretation. We apologize for the missing explanation. Corrected.

b) *Section 3.3, as already said above, it is not 100% clear what the authors refer to with trial-to-trial variability. Is each dt considered a trial? If yes, it would be helpful to spell this out in the text.*

We now use Fano factor instead of trial-to-trial variability and in this particular instance we try to be very explicit about how we calculate it.

c) *Section 4, at the beginning of the discussions, the authors claim that "populations in the cortex that transmit information by their summed activity can be thought of as low noise information channels". Although I believe this is true in theory, it would be helpful to know if this strategy is employed in practice by the brain (refer to comment 2 above).*

We hope that our response to Comment 2 also responds to this comment. Moreover, we now dropped this formulation from the discussion.

11. *Figures:*

a) *Fig 1, panel B) it'd be useful to have a title for the colorful legend, and it would be useful to invert the scale (i.e. start from high at the top to low at the bottom). Also, are the two subplots related to excitatory and inhibitory cells?*

We added the legend title and inverted the order of the labels (also in Fig. 5A). Both subplots are the same functions at different locations and scales. We now explain this in the caption.

b) *Fig 1, panel B) and F), Fig 2, Fig 3E), Fig 4, Fig 5A): it'd be useful to have a title for the colorful legends so as to avoid needing to search for that in the figure caption.*

Done.

Response to Referee 2

1. *On pg. 4, to compute the mean number of pre-synaptic external neurons for each neuron in the recurrent network, the authors use formula* $N_{\mathrm{ext}}\frac{100\%}{P_{\mathrm{ext}}}$*. But on pg. 11 they use formula* $N_{\mathrm{ext}}P_{\mathrm{ext}}$*. First of all, I would recommend that the notation be streamlined – is the external population denoted as* n_{ext} *or* N_{ext}*? Similarly, is* P_{ext} *given in % or has a value between [0,1]? Secondly, I would really appreciate it if the authors could clarify if what they have stated on pg. 4 and pg. 11 are two different ideas or one of them is incorrectly computed.*

We now denote all population counts with lower case letter only (n_{ext}, n_{exc}, n_{inh}). P_{ext} has a value between 0 and 1. We edited the text to make this consistent across the manuscript. We apologize for the previous incoherence. Formula on pg. 4 represents something else, as is now explained in the text. $\frac{n_{\text{ext}}}{P_{\text{ext}}}$ does not represent the number of pre-synaptic neurons, instead, $\frac{1}{P_{\text{ext}}}$ is a scaling factor which accounts for the lower number of pre-synaptic neurons with lower values of P_{ext}.

2. *Eq. 48 seems inconsistent with the text right before it. Is the equation $c_1 \exp(c_2 x)$ or $c_1 \exp(-c_2 x)$? Also, I tried to solve the integral in Eq. 48 and did not get the same answer as the authors. It would be helpful for me to see the calculation – but I am not asking for this to be in the paper.*

   The sign in Eq. 48 was incorrect. The correct formula is $c_1 \exp(c_2 x)$, as given in the text (increasing output with increasing output). Below, we provide the solution of the integral (here we replaced λ_{ext} with λ for easier notation):

$$\int_{-\infty}^{\infty} \frac{c_1}{\sigma\sqrt{2\pi}} \exp\left[-\frac{(x-\lambda)^2}{\sigma^2} + c_2 x\right] \mathrm{d}x =$$
$$= \frac{c_1}{\sigma\sqrt{2\pi}} \int_{-\infty}^{\infty} \exp\left[-\frac{1}{\sigma^2}x^2 + \left(c_2 + \frac{2\lambda}{\sigma^2}\right)x - \frac{\lambda^2}{\sigma^2}\right] \mathrm{d}x =$$
$$= \frac{c_1}{\sigma\sqrt{2\pi}} \int_{-\infty}^{\infty} \exp\left\{-\left[\frac{x}{\sigma} - \left(\frac{c_2\sigma}{2} + \frac{\lambda}{\sigma}\right)\right]^2 + \frac{c_2^2\sigma^2}{4} + c_2\lambda\right\} \mathrm{d}x =$$
$$= \frac{c_1}{\sigma\sqrt{2\pi}} \exp\left(\frac{c_2^2\sigma^2}{4} + c_2\lambda\right) \int_{-\infty}^{\infty} \sigma \exp\left(-z^2\right) \mathrm{d}x =$$
$$= \frac{c_1}{\sqrt{2}} \exp\left[\frac{c_2}{4}\left(\sigma^2 + 4\lambda\right)\right]$$

   Thus we obtained the expression provided in Eq. 48 of the previous version of the manuscript (with a different sign in front of λ due to changing the sign in front of c_2). We note that in the denominator of the exponential, we should have used $2\sigma^2$ instead of σ_2 to properly represent the Gaussian distribution. After this substitution, we obtain the formula:

$$\int_{-\infty}^{\infty} \frac{c_1}{\sigma\sqrt{2\pi}} \exp\left[-\frac{(x-\lambda)^2}{2\sigma^2} + c_2 x\right] \mathrm{d}x = \frac{c_1}{2} \exp\left[\frac{c_2}{2}\left(\sigma^2 + 2\lambda\right)\right],$$

   which is in the current version of our manuscript.

Minor concerns

1. *Eq.7 – did you mean g_{inh}?*

   Yes, we did. Corrected.

2. *Please use consistent formats for probability of connection – as stated before two different formats have been used for , please be consistent and also keep it consistent with .*

   We now consistently report the probabilities in the range between 0 and 1.

3. *In Table 1, g has been used to denote scaling factor for the inh. synapse amplitude but then g is the variable that has been defined. Check for consistency. Also, g in Eq. 34 is used to symbolize the gain of the system. Please check that variables are not repeated.*

   We renamed the scaling factor as α. We denote the gain of the system as g, because this is consistent with the definition $g = \mu'(\lambda_{\text{ext}})$.

4. *On pg. 13, the last paragraph states that a 7th-degree polynomial was fit – can you explain why? Is this borrowed from previous literature or have other degrees been tested and this was the best fit?*

   It was purely our observation that 7-th degree polynomial captures well the course of the function without producing substantial oscillations. To avoid any confusion, we now use linear interpolation. The effect on the results in negligible.

5. *In Eq. 21 and in a few other places, I think the authors mean to refer to* n_{ext}, n_{exc}, n_{inh} *as defined on pg. 3. Please check notation.*

   Corrected.

6. *For Eq. 32,* σ *has been defined but not* μ'*. Define both.*

   In the new version we defined μ and μ', and also removed the lower index ext, as it does not belong there.

7. *In Eq. 37, I might have misunderstood this, but there is a constant term of* $\frac{\exp(W_0)}{\sqrt{e}}$ *missing. Is the value of this constant 1?*

   The terms were missing indeed. We now include them. However, any constant term has no effect on the final result, as it will appear in the multipliers λ_1 and λ_W.

8. *In the results subsection of 3.2, the scaling value of has been chosen from 15 to 25. Cite a reference that justifies these values.*

   We added justification and references.

9. *In Fig.1,*

   (B) why is this plot randomly cut in the middle?

   (C) what is the black line? Add in legend.

   The plot is cut in the middle due to scaling purposes. This is now explained in the caption. The black line in panel (C) represents a feedforward network without any recurrent connections ($a_{\text{rec}} = 0$). This is now explained in the caption and added in the legend.

10. *On pg. 11 in the last paragraph, the authors refer to the mean Fano factor in Fig.4D-F, but I think they mean Fig.4G-I. Also, maybe they can comment on the gain increasing with which is in fact shown in Fig.4D-F.*

    Thank you for bringing this to our attention. We corrected the panel label and also comment on the panels D-F.

11. *My interpretation of Fig.3A and B is that A shows synaptic current from the background activity + action potentials in the background activity and B shows action potentials in the neuronal network that are evoked by the background activity. If this is correct, please modify the text in the caption because it is slightly confusing as is presently written.*

    In A we show the cost arising from all excitatory synaptic currents. In the Figure we do not distinguish whether these currents originate from the recurrent synapses or from the background input. We changed the wording from background activity to background input and added a reference to the background input equation. We did not include the cost of action potentials of the background activity. This was mentioned and justified in the Discussion. We moved this information in the results section, where we believe it fits more appropriately and will help avoid confusion.

References

D. Attwell and S. B. Laughlin. An Energy Budget for Signaling in the Grey Matter of the Brain. *J. Cereb. Blood Flow Metab.*, 21(10):1133–1145, 2001. doi: 10.1097/00004647-200110000-00001.

S. Panzeri, M. Moroni, H. Safaai, and C. D. Harvey. The structures and functions of correlations in neural population codes. *Nat. Rev. Neurosci.*, 23(9): 551–567, 9 2022. ISSN 1471-003X. doi: 10.1038/s41583-022-00606-4.

G. Tkačik, J. S. Prentice, V. Balasubramanian, and E. Schneidman. Optimal population coding by noisy spiking neurons. *Proc. Natl. Acad. Sci. U.S.A.*, 107(32):14419–14424, 8 2010. ISSN 0027-8424. doi: 10.1073/pnas.1004906107.

P. Vetter, A. Roth, and M. Häusser. Propagation of Action Potentials in Dendrites Depends on Dendritic Morphology. *J. Neurophysiol.*, 85(2):926–937, 2 2001. ISSN 0022-3077. doi: 10.1152/jn.2001.85.2.926.

Y. Zerlaut, S. Chemla, F. Chavane, and A. Destexhe. Modeling mesoscopic cortical dynamics using a mean-field model of conductance-based networks of adaptive exponential integrate-and-fire neurons. *J. Comput. Neurosci*, 44(1): 45–61, 2 2017. ISSN 0929-5313. doi: 10.1007/s10827-017-0668-2.
