# Supplementary material for: Shared input and recurrency in neural networks for metabolically efficient information transmission
Source: PLoS Comput Biol. 2024 Feb 23;20(2):e1011896. doi: 10.1371/journal.pcbi.1011896 (PMC10917264; doi:10.1371/journal.pcbi.1011896)
Supplement: Attachment — Submitted filename: Barta Kostal R1.pdf [file pcbi.1011896.s009.pdf]

Dear Authors,

I am pleased with the revised version. I like the new extended sect. 2.1, which introduces the main points of the problem; and the extended explanations about what the network represents and its interpretation + putative function. The last addition, given by the optimal decoder, is also insightful. Also, thank you for the small improvements throughout the methods; they make everything clearer and more readable; and the improvements on the figures. I am happy to recommend this new version.

I would simply ask the authors to fix/clarify a couple of sentences that I found slightly confusing:

1) "In the case of our simple linear the Fisher information (Eq. 14) is.."
(just above eq (16))

2) "We performed dimensionality reduction of the output across all stimuli by principal component analysis and used the first 500 principal components. We used 500, because the increase in information-metabolic efficiency for higher number of components is small, and the sampling bias is still relatively small (S5 Fig). To deal with the remaining sampling bias we calculated the information-metabolic efficiency with the Jimbo-Kunisawa for different numbers of trials and performed the quadratic extrapolation method to estimate the unbiased information-metabolic efficiency (Strong et al., 1998; Panzeri et al., 2007)."

At first, I found this sentence to be very confusing, possibly because you didn't say what you were doing PCs for. Then I understood that, to reduce the effect of sampling bias on info estimates, you are doing an analysis on the PCs, rather than the actual output, and then use Strong's quadratic extrapolation.
Not sure what is best here; I think it'd be useful to have a sentence describing the problem and maybe you could also:
a) either shorten here and expand in methods (like "we used various techniques to properly estimate entropic quantities (see methods sect. 4.4)"
b) or focus on the results in this section and move the technical bit altogether to the methods section 4.4?

3) "Overall, the results remain qualitatively very similar to the information-metabolic efficiency calculated from the summed activity. However, we found the increase in information-metabolic efficiency from using high-dimensional output is the largest for higher values a_rec and P_ext."

You don't mention which figure this refers to (Fig 6A-B right?), and it's useful to briefly re-iterate the results and point to the previous figures resembling it, rather than simply saying "..results remain qualitatively very similar to..".

4) "Information from a neural population carries more information about the stimulus than a single neuron."
(beginning of discussion)
