# Supplementary material for: Shared input and recurrency in neural networks for metabolically efficient information transmission
Source: PLoS Comput Biol. 2024 Feb 23;20(2):e1011896. doi: 10.1371/journal.pcbi.1011896 (PMC10917264; doi:10.1371/journal.pcbi.1011896)
Supplement: Attachment — Submitted filename: response.pdf [file pcbi.1011896.s010.pdf]

Dear Editor, Dear Reviewers,

We would like to thank the reviewers for their positive assessment of our work, and we are happy they were pleased with the changes. We addressed the remaining unclarities pointed out by **Reviewer 1**, in particular:

1. *"In the case of our simple linear the Fisher information (Eq. 14) is.." (just above eq (16))*

   Corrected to "linear system".

2. *"We performed dimensionality reduction of the output across all stimuli by principal component analysis and used the first 500 principal components. We used 500, because the increase in information-metabolic efficiency for higher number of components is small, and the sampling bias is still relatively small (S5 Fig). To deal with the remaining sampling bias we calculated the information-metabolic efficiency with the Jimbo-Kunisawa for different numbers of trials and performed the quadratic extrapolation method to estimate the unbiased information-metabolic efficiency (Strong et al., 1998; Panzeri et al., 2007)."*

   *At first, I found this sentence to be very confusing, possibly because you didn't say what you were doing PCs for. Then I understood that, to reduce the effect of sampling bias on info estimates, you are doing an analysis on the PCs, rather than the actual output, and then use Strong's quadratic extrapolation.*

   *Not sure what is best here; I think it'd be useful to have a sentence describing the problem and maybe you could also:*

   a) *either shorten here and expand in methods (like "we used various techniques to properly estimate entropic quantities (see methods sect. 4.4)"*
   b) *or focus on the results in this section and move the technical bit altogether to the methods section 4.4?*

   We modified the text in Results to make it more self-sufficient and put more details in the Methods sections.

3. *"Overall, the results remain qualitatively very similar to the information-metabolic efficiency calculated from the summed activity. However, we found the increase in information-metabolic efficiency from using high-dimensional output is the largest for higher values $a_{\mathrm{rec}}$ and $P_{\mathrm{ext}}$."*

   *You don't mention which figure this refers to (Fig 6A-B right?), and it's useful to briefly re-iterate the results and point to the previous figures resembling it, rather than simply saying "..results remain qualitatively very similar to...".*

   We fixed the missing reference to Fig. 6. Additionally, we now also specifically describe what is same and what is different in comparison to the previous results.

4. *"Information from a neural population carries more information about the stimulus than a single neuron." (beginning of discussion).*

We changed the sentence to the following: “One of the benefits is that by considering the signal from many neurons, it is possible to decrease the noise inherent to rate coding spiking neurons, and thus increase the information carried by the system.”

**Reviewer 2** did not request any additional changes. Additionally, we would like to point out the additional changes in the manuscript:

1. Due to small correction in calculations, Fig. 6 has been slightly modified, leading only to minor quantitative differences.

2. In the second version of the paper, we used a slightly different value of a cost of action potential, than in the first version, as explained in the previous response to Reviewers. We forgot to update all relevant figures. Therefore, there are minor quantitative changes in the following:

   - Fig. 3
   - Fig. 5, panels C-H, and the corresponding Table 1.
   - S3 Fig. and S4 Fig.

We hope that you will find the current version of the manuscript suitable for publication in *PLoS Computational Biology.*

Sincerely,
Tomas Barta
