# Supplementary material for: Shared input and recurrency in neural networks for metabolically efficient information transmission
Source: PLoS Comput Biol. 2024 Feb 23;20(2):e1011896. doi: 10.1371/journal.pcbi.1011896 (PMC10917264; doi:10.1371/journal.pcbi.1011896)
Supplement: Attachment — Submitted filename: Review.docx [file pcbi.1011896.s007.docx]

The paper analyzes the effect of two factors on the information-metabolic efficiency of a recurrent neuronal network. The first factor is the external input from thalamocortical synapses which introduces neuronal pairwise correlations in the network. The second factor is the presence of inhibitory feedback which can reduce these correlations. By studying the balance between these factors, the authors find that both the shared input and inhibitory feedback play a role in increasing information-metabolic efficiency of the system.

This is an interesting paper but I have some concerns with regards to the writing and some of the calculations.

1. On pg. 4 to compute the mean number of pre-synaptic external neurons for each neuron in the recurrent network, the authors use formula $n_{ext}\frac{100\%}{P_{ext}}$ . But on pg. 11 they use formula $N_{ext}*P_{ext}$.
   First of all, I would recommend that the notation be streamlined – is the external population denoted as $n_{ext}$ or $N_{ext}$? Similarly, is $P_{ext}$ given in % or has a value between [0,1]?
   Secondly, I would really appreciate it if the authors could clarify if what they have stated on pg. 4 and pg. 11 are two different ideas or one of them is incorrectly computed.
2. Eq. 48 seems inconsistent with the text right before it. Is the equation $c_{1}exp(c_{2}x)$ or $c_{1}exp(-c_{2}x)$? Also, I tried to solve the integral in Eq. 48 and did not get the same answer as the authors. It would be helpful for me to see the calculation – but I am not asking for this to be in the paper.

Minor concerns:

1. Eq.7 – did you mean $g_{inh}$?
2. Please use consistent formats for probability of connection – as stated before two different formats have been used for $P_{ext}$, please be consistent and also keep it consistent with $P_{rec}$.
3. In Table 1, $g$ has been used to denote scaling factor for the inh. synapse amplitude but then ‘g’ is the variable that has been defined. Check for consistency. Also, $g$ in Eq.34 is used to symbolize the gain of the system. Please check that variables are not repeated.
4. On Pg.13, the last paragraph states that a 7^th^-degree polynomial was fit – can you explain why? Is this borrowed from previous literature or have other degrees been tested and this was the best fit?
5. In Eq.21 and in a few other places, I think the authors mean to refer to $n_{ext}, n_{exc}, n_{inh}$ as defined on pg. 3. Please check notation.
6. For Eq.32, $\sigma$ has been defined but not $\mu'$. Define both.
7. In Eq.37, I might have misunderstood this, but there is a constant term of $\frac{\exp\left( w_{0} \right)}{\surd e}$ missing. Is the value of this constant 1?
8. In the results subsection of 3.2, the scaling value of $g$ has been chosen from 15 to 25. Cite a reference that justifies these values.
9. In Fig.1,
   (B) why is this plot randomly cut in the middle?
   (C) what is the black line? Add in legend.
10. On pg. 11 in the last paragraph, the authors refer to the mean fano factor in Fig.4D-F, but I think they mean Fig.4G-I. Also, maybe they can comment on the gain increasing with $P_{ext}$ which is infact shown in Fig.4D-F.
11. My interpretation of Fig.3A and B is that A shows synaptic current from the background activity + action potentials in the background activity and B shows action potentials in the neuronal network that are evoked by the background activity. If this is correct, please modify the text in the caption because it is slightly confusing as is presently written.
